# Barriers to screening of breast and cervical cancer among women in remote villages of Karnataka: an analysis using the Health Belief Model

**DOI:** 10.3332/ecancer.2025.1980

**Published:** 2025-08-29

**Authors:** Mayank Chhabra, Somika Meet, Gandhar Tendulkar, Kunal Oswal, Milan Toraskar, Sai Murali, Bharat Kumar Sarvepalli, Sripriya Rao, Ramachandran Venkataramanan, Yogesh Jain

**Affiliations:** 1 Department of Women Wellness and Early Detection, Karkinos Healthcare Private Limited, Mumbai 400021, Maharashtra, India; 2 Department of Screening and Early Detection, Karkinos Healthcare, Mumbai 400021, Maharashtra, India; 3 Department of Women Wellness, Karkinos Healthcare, Hyderabad 500001, Telangana, India; 4 Department of Clinical Research, Karkinos Healthcare, Mumbai 400021, Maharashtra, India; 5 Distributed Cancer Care Network and Chief Growth Officer, Women Wellness, Karkinos Healthcare Private Limited, Bengaluru 560001, Karnataka, India; 6 Chief Executive Officer and Founder, Karkinos Healthcare, Mumbai 400021, Maharashtra, India; 7 Non-Communicable Diseases, Karkinos Healthcare, Bilaspur 495001, Chhattisgarh, India; a https://orcid.org/0009-0007-8677-0979; * Joint first authors

**Keywords:** breast cancer, cervical cancer, cancer screening, Health Belief Model, rural health, barriers, community-based programs, India

## Abstract

**Background:**

Cancer remains a significant public health challenge, being the second leading cause of death in urban areas and the fourth in rural regions of India. The estimated 1.15 million new cancer cases in 2018 are projected to double by 2040. Despite the critical importance of early detection, cancer screening rates in rural India remain alarmingly low. This study investigates barriers to breast and cervical screening among women in remote villages of Karnataka using the Health Belief Model (HBM) as a theoretical framework.

**Methods:**

A community-based screening program for oral, breast and cervical cancer, was implemented in three taluks of Chikkaballapur district, Karnataka, from September to November 2021. Quantitative data from 4,974 screened women were complemented by qualitative interviews with 292 women who did not consent to screening, particularly for breast and cervical cancer. Interviews were guided by HBM constructs perceived susceptibility, severity, barriers, benefits, cues to action and self-efficacy and analysed thematically.

**Results:**

Out of the 4,974 women who participated in screening clinics, less than 10% consented to clinical breast examination and none to cervical screening. Major barriers to screening included socio-cultural factors (stigma, lack of awareness, peer pressure), economic constraints (work priorities and financial insecurity), psychological barriers (fear of outcomes and lack of healthcare trust) and physical challenges (accessibility and seasonal constraints). Fear of treatment outcomes and financial implications were prominent psychological deterrents. Mitigation strategies were noted to address these barriers, including awareness campaigns, flexible camp timings and local stakeholder engagement.

**Conclusion:**

Addressing barriers to cervical and breast cancer screening requires a holistic, community-centred approach informed by theoretical models like HBM. Sustainable interventions must prioritise awareness, accessibility and affordability to bridge critical healthcare gaps and reduce the burden of cancer in rural India.

## Introduction

Cancer remains a significant public health challenge, being the second leading cause of death in urban areas and the fourth in rural regions of India. The estimated 1.15 million new cancer cases in 2018 are projected to double by 2040 [1]. Among women, breast cancer (26.6%), cervical cancer (17.7%), ovarian cancer (6.6%), lip and oral cavity cancer (5%) and colorectal cancer (3.7%) are the most prevalent [2].

Despite the growing burden of cancer, the status of screening in India remains critically low [3]. National Family Health Survey-5 (2019–21) reveals that the percentage of women in Karnataka ever screened for breast, cervical and oral cancers was just 0.2%, 0.5% and 0.4%, respectively [4]. Moreover, the projected incidence of cancer cases in Karnataka is expected to reach 51,437 by 2025 [5] owing to an increase in tobacco/alcohol abuse and lifestyle changes [6]. Evidence suggests that early detection of breast and cervical cancers can significantly improve survival rates. However, several economic, psychosocial and cultural barriers impede the uptake of screening services across the country [7].

Understanding women’s attitudes, beliefs and perceptions about screening is fundamental to developing effective and targeted screening programs [8]. The Health Belief Model (HBM), a widely applied theoretical framework, provides valuable insights into health behaviours through six constructs: perceived susceptibility, perceived severity, perceived benefits, perceived barriers, self-efficacy and cues to action [9]. Beliefs about perceived benefits of health promotion are weighted against perceived barriers while cues to action affect one’s views about barriers or benefits. While HBM does not explain causal relationships, it can provide a framework for improving cancer screening uptakes and outcomes [10].

Public health practitioners can design more impactful interventions by leveraging the HBM framework to analyse and address the attitudes and perceptions of specific target populations. Karkinos Healthcare Private Limited implemented a community-based cancer screening program in the Chikkaballapur district of Karnataka in 2021. This study aims to present the outcomes of this initiative and explore the barriers to screening uptake among participants using HBM. While participation in oral cancer screening was high, the uptake for breast and cervical cancer screenings was low. Therefore, this study specifically aims to explore the barriers to breast and cervical screening uptake.

## Methodology

### Project site

**Figure 1.**
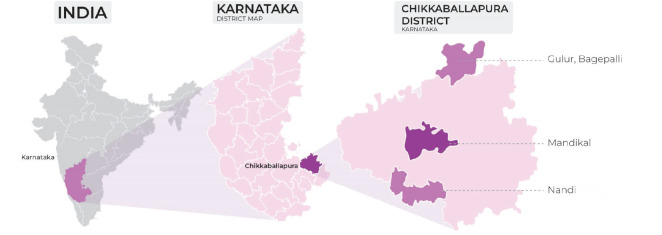
Intervention sites for cancer screening program.

A community screening program for cancer was conducted from September to November 2021 in Chikkaballapur district of Karnataka, India. It has a population of 12,55,104 and is situated in the southern parts of the state [11]. Three talukas (blocks), Nandi, Mandikal and Bagepalli were selected as the intervention rural sites. Figure 1 depicts the intervention sites in Karnataka.

### Screening for common cancers

Awareness and education campaigns about risk factors and symptoms of common cancers were conducted by trained medical staff before the screening intervention. It also included Accredited Social Health Activist-based home visits, flyer distribution, audio announcements, early screening rallies and local newspaper coverage. A total of 312 awareness sessions in 74+ villages were undertaken to mobilise the community.

Population-based screening clinics and health education programs were organised at various local governmental bodies, including public health centres, sub centres, anganwadis, panchayati bhavans, schools and so on. Screening for oral, breast and cervical cancer was done through oral visual examination, clinical breast examination (CBE) and human papillomavirus test (HPV), respectively.

Women in the age group of 35–60 years and those who provided informed consent were screened. Women who were pregnant or on their menstrual cycle were excluded from breast and cervical screening.

### Data collection and analysis

A mixed-methods study design was adopted, using quantitative data to identify the screening rates and results. All screening data were recorded through a customised digital application.

Women who did not consent to undergo breast and cervical screening were included in the study. A total of 292 participants were selected purposively and were interviewed to understand their barriers to participation.

A HBM was used to understand the barriers to breast and cervical screening. The semi-structured interview guide (Table 1) was designed according to the domains of the model, including perceived susceptibility, perceived severity, perceived barriers, perceived benefits, cues to action and health motivation and self-efficacy. Additionally, demographic details were also captured. The interview was conducted by trained local volunteers. The screening data were analysed in MS Excel and thematic analysis was done for the interviews conducted.

### Ethical consideration

Informed consent was taken before screening. The consent forms were available in local languages (Hindi, Telugu and Kannada) and included consent for all screenings. The study was approved by the Sri Durgamba Independent Ethics Committee (IEC) dated 11/09/2021.

## Results

A total of 4,974 women were screened in three rural sites, where the majority of them were from Nandi (43.2%), followed by Mandikal (33.5%) and Bagepalli (23.3%). Table 2 describes the socio-demographic factors of the participants.

52.4% of the women who underwent screening were housemakers while 37% were farmers. Table 3 shows the screening results in participants in the outreach clinics.

### Barriers to breast and cervical cancer screening in a community setting

Several challenges and barriers existed in the community screening clinics. The major barriers were classified into socio-cultural, economic, psychological and physical barriers. Sub-themes were created thereafter. Table 4 shows the barriers and challenges faced by women.

The social stigma associated with cervical and breast screening emerged as a major concern among women. Additionally, a lack of awareness of the importance of early detection hindered participation. No participant consented to HPV screening due to fear of pain during procedure and feeling of embarrassment. Peer pressure and language barriers further complicated outreach efforts, while poor family support added to the reluctance of individuals to seek screening services.

*‘My mother-in-law advised me against visiting the camp as she heard our private videos will be created.’ (32-year-old participant)*

*‘I have been having nouvuu (pain) in my breasts for the last 6 months, but my husband’s family does not allow me to seek treatment.’ (30-year-old participant)*

*‘My body is absolutely fine. I am in no need of any checkups.’ (55-year-old participant)*

Many individuals faced poor financial support and a lack of insurance coverage, which discouraged them from attending screenings or pursuing treatment. Additionally, work or priorities often took precedence over health, leaving little time or willingness to attend screening camps.

*‘We are daily wagers. If we do not turn up for work, we will lose one day’s salary which we cannot afford. We need to feed our families.’ (35-year-old participant)*

*‘Who will take care of us if we get sick? We have no money.’ (41-year-old participant)*

*‘I’m scared they’ll find something serious, and then I’ll need surgery or medicines. What will happen to my family if I can’t work anymore?’*

The psychological barriers were observed in the form lack of trust in the healthcare system. Concerns related to the procedures and outcomes were also reported, indicating fear and uncertainty regarding screening results.

*‘If I go to a hospital, they will take out my organs. I am very afraid of seeing a doctor.’ (59-year-old participant)*

*‘The test is free, but if something is found, I’ll need money for treatment. How will we afford that when we barely make enough to eat?’ (41-year-old participant)*

*‘What if they say I have cancer? It’s better not to know. I would rather live in peace without worrying about it.’ (60-year-old, participant)*

Physical barriers significantly impacted participation, particularly due to external factors such as agricultural or festival seasons when individuals prioritised other responsibilities. Additionally, weather conditions, such as heavy rains, made it difficult for people to travel and poor accessibility to camp locations further restricted attendance.

*‘The camp was too far from our village. We don’t have buses, and hiring an auto is expensive. Only a few of us could go.’ (45-year-old, participant)*

Mitigation strategies were identified during the interviews and implemented during the program to overcome the challenges of the seamless conduct of community screening clinics and increase participation. The cues to action to overcome key challenges are listed in Table 5.

## Discussion

This study identified several barriers to cancer screening in rural community settings, highlighting the multifaceted challenges faced by women. Socio-cultural factors, including stigma associated with screening procedures, lack of awareness and poor family support, emerged as significant barriers. Economic challenges, such as work priorities and financial insecurity, further discouraged participation. Psychological barriers were prominent, including a lack of trust in the healthcare system and fear of screening outcomes or costs. Additionally, physical factors like inaccessible camp locations, adverse weather and agricultural or festival priorities compounded the problem.

Psychosocial barriers remain the same over the years. Psychological barriers such as fear of screening procedure and fear of being diagnosed with cancer, among other factors like lack of awareness, cultural beliefs, embarrassment, financial and health system-related factors, were identified in a study conducted in 2020 in Tamil Nadu [7]. Another study conducted among Indian rural women in Maharashtra also identified lack of awareness and lack of education as barriers to breast and cervical cancer screening [12].

Such challenges and barriers can lead to missed or underreported cases of pre-existing health conditions and risk factors, potentially representing the tip of the iceberg in understanding the true burden of cancers. These findings accentuate the need for tailored interventions, including community outreach, counselling, flexible scheduling, financial aid awareness and local stakeholder involvement, to address these barriers and improve participation in cancer screening programs.

Drawing insights directly from the experiences of individuals who declined participation in screening camps, we identified the following mitigation strategies such as addressing lack of awareness through community outreach and counseling; overcoming psychological barriers by involving local influencers and offering family counseling; accommodating economic constraints with flexible timings; raising awareness about financial aid and government schemes to tackle costs; aligning camp schedules with agricultural and festival seasons; and mitigating physical barriers by establishing local outreach clinics. Importance of social support networks, role of community health workers, financial aid, culturally sensitive care and logistic improvements were identified as crucial enablers to cancer screening by participants of a study that was conducted in low-income communities [13].

The HBM is useful in predicting barriers to cancer screening among women. Studies have found that perceived barriers are a significant predictor of breast self-examination and mammography frequency. Perceived benefits and self-efficacy also play important roles in predicting screening behaviours. Women with higher perceived susceptibility to breast cancer, often due to family history or previous abnormal results, are more likely to undergo regular screenings [14].

Studies have shown that HBM-based education can positively influence women's beliefs and attitudes toward cervical cancer screening. Hence, HBM can be adopted while planning screening interventions where these barriers can be addressed through health promotion activities. To improve cancer screening rates, interventions should focus on reducing barriers, increasing perceived benefits, enhancing self-efficacy and improving overall knowledge about cancer and screening procedures [15].

The HBM remains valuable for designing health interventions; however, it has limitations. Studies show that the model's predictive capacity is low, with an average *R*^2^ <0.21 [16]. Perceived barriers and benefits are the strongest predictors, while perceived severity is the weakest [17]. The model's effectiveness is limited by its abstract nature, emphasis on patient rationality and lack of clear rules for combining variables [16]. Despite these limitations, HBM-based interventions remain worth exploring, as they offer a structured approach to addressing the gap between theoretical understanding and practical application. By customising strategies that account for specific barriers and emphasise the benefits of early detection, such interventions can play a pivotal role in enhancing health outcomes, even in resource-constrained settings.

## Conclusion

To address the burden of non-communicable diseases in rural India, it is crucial to understand the socio-cultural, economic, psychological and physical barriers that hinder screening uptake. Integrating community-tailored screening models with robust health systems and targeted awareness initiatives can promote early detection and timely intervention. By leveraging frameworks like the HBM, interventions can be better designed to address barriers, enhance trust and improve participation. These strategies can ultimately reduce disease burden, improve health outcomes and contribute to equitable healthcare access in resource-constrained settings.

## Conflicts of interest

The authors declare that they have no conflicts of interest.

## Funding

This research did not receive any specific grant from funding agencies in the public, commercial, or not-for-profit sectors.

## Author contributions

Mayank Chhabra (MC), Somika Meet (SM), Gandhar Tendulkar (GT), Kunal Oswal (KO), Milan Toraskar (MT), Bharat Kumar Sarvepalli (BS), Sripriya Rao (SR), R Venkataramanan (RV), Yogesh Jain (YJ). Conception and design: MC, KO, SR, RV. Administrative support: BS, RV. Collection and assembly of data: MC, SM, GT, MT. Data analysis and interpretation: MC, SM, GT. Technical inputs and manuscript review: YJ, BS, KO. Proofreading: YJ, KO, BS, RV. Manuscript writing: All authors. Final approval of manuscript: All authors. Accountable for all aspects of the work: MC, SM, GT, KO.

## Figures and Tables

**Table 1.** Semi-structured interview guide following the HBM.

Domain	Topics
Perceived susceptibility	What are the risk factors and symptoms of breast and cervical cancers?
Perceived susceptibility	What will you do if diagnosed with cancer?
Perceived barriers	What stops you from uptaking screening?
Cues to action	How do you think we can improve screening behaviour among healthy individuals?
Perceived benefit	Do you think screening is useful for early detection of cancer?
Self-efficacy	What internal factors prevent you from getting screened?

**Table 2.** Socio-demographic factors of the participants.

Demographics parameter	Variables	Frequency (%) *N* = 4,974
Age	35–44 years	2,451 (49.7)
	45–54 years	1,370 (27.3)
	55–65 years	1,153 (23)
Occupation	Agriculture & daily wagers	1,838 (37)
	Manufacturing & services	130 (2.6)
	Housemakers	2,607 (52.4)
	Others	399 (8)

**Table 3.** Distribution of screening results of the participants in the outreach clinics.

Screening	Frequency (%)
Screened for oral cancer	4,974 (100%)
Screened for breast cancer	491 (9.9%)
Screened for cervical cancer	0 (0%)
**Screening results (*N* = 4,974)**	**Frequency (%)**
Oral potentially malignant disorders (OPMDs)	459 (9.2)
CBE positive*	28 (5)
HPV positive**	Unavailable

* CBE positive out of total screened for CBE

** Cervical cancer data is unavailable due to a lack of participant consent for testing

**Table 4.** Barriers/challenges faced by women who did not participate in community screening clinics.

HBM constructs	Themes	Subthemes
Perceived barriers, perceived susceptibility, self-efficacy	Socio-cultural	Social stigma
		Lack of awareness and language barrier
		Peer pressure
		Poor family support
Perceived barriers, perceived benefits	Economic	Poor financial support
		Work priority
Perceived barriers, perceived severity	Psychological	Lack of trust
		Fear of procedures
		Treatment consequences
Perceived barriers, self-efficacy	Physical barriers	Agricultural/festival season
		Weather conditions
		Poor accessibility
Perceived susceptibility	Socio-cultural	Religious beliefs

**Table 5.** Key challenges/barriers for not visiting rural community-based screening programs and mitigation strategies (cues to action) to overcome these challenges.

Key challenges	Mitigation strategies
Lack of awareness/poor understanding	• Local awareness through community outreach and campaign • Continuous counselling about camp objectives
Psychological (Lack of trust and fear of procedures)	• Involving local influencers/women activists or village panchayat heads • Family counselling
Economic (Work priority)	• Flexible screening camp timing (Early morning and late evening slots)
Lack of financial support/ Treatment and screening cost	• Awareness about: • Financial assistance with governmental health schemes (PMJAY, Ayushman Bharat) • Govt aid referral schemes for treatment
Agricultural (Harvesting season)/festival season	• Optimize the camp and outreach calendar based on the local situation and seasons
Physical factors (Rain, distance)	• Setting up outreach clinics in nearby health facilities or Anganwadi centre or school premises
